# Life Cycle Assessment of the Construction and Demolition Waste Recovery Process

**DOI:** 10.3390/ma18204685

**Published:** 2025-10-13

**Authors:** Mateusz Malinowski, Zuzanna Basak, Stanisław Famielec, Arkadiusz Bieszczad, Sabina Angrecka, Stanisław Bodziacki

**Affiliations:** 1 Department of Bioprocess Engineering, Power Engineering and Automation, Faculty of Production and Power Engineering, University of Agriculture in Kraków, 30-149 Kraków, Poland; 2 Department of Rural Building, Faculty of Environmental Engineering and Land Surveying, University of Agriculture in Kraków, 31-120 Kraków, Poland; 3 Department of Microbiology and Biomonitoring, University of Agriculture in Kraków, 30-059 Kraków, Poland

**Keywords:** circular economy, waste treatment facilities, waste material composition, material recovery, environmental impact assessment

## Abstract

Effective recovery of materials from construction and demolition waste (CDW) remains a major problem and a real challenge in terms of implementing the circular economy. In many countries, this waste is landfilled due to the lack of modern technological lines for its recovery and recycling, including the sorting of materials suitable for reuse. Understanding the environmental impact of the CDW treatment process is important as it constitutes the final stage of building life cycle assessment and the basis for eco-design of construction processes. In addition, the recovered materials can be used as raw materials for construction, thereby closing the waste loop and aligning with the circular economy concept. The purpose of this study is to compare the environmental impact of three different CDW recovery technologies in order to identify the optimal option. The analysis was performed using the life cycle assessment (LCA) methodology, SimaPro 8.1 software, and the Ecoinvent v3.8 database. 1 Mg of processed CDW was adopted as the functional unit. It was found that the process of recovering materials from CDW allows for sorting over 13% of materials for recycling and approx. 40% of raw materials for reuse (stone aggregates). The conducted analyses showed that all three installations exert a negative impact on the environment. Solution No. 2 had the lowest total environmental impact (15.96 Pt) under the assumptions and datasets used in this study, presenting average electricity and fuel consumption and average weight of sorted materials for recycling. Installation No. 3, which sorts the largest volume of materials for recycling, also used the most electricity; therefore, it could not be considered as the solution with the minimal overall environmental impact. The research revealed that the treatment of CDW in a crusher, applied at all installations, is the process stage resulting in the greatest environmental pressure (16.92 Pt). The high level of sorted recyclable waste enabled a relatively low carbon footprint for processes No. 2 and No. 3, 18.7 and 17.6 kg CO_2_ eq, respectively (more than four times lower than for installation No. 1). Future analyses should focus on optimizing the CDW recovery process by avoiding the use of impact crushers, as adding more waste sorting equipment does not significantly enhance environmental benefits.

## 1. Introduction

The implementation of new, innovative production methods, low-waste technologies, and emission reduction measures (into the air, soil, and water) allows for a decrease in material and natural resource consumption, thereby reducing the negative environmental impact of the economy [1,2]. All of these activities are an integral part of the sustainable development strategy, Industry 4.0, and the circular economy [3]. To support the circular economy, policymakers have a whole set of tools at their disposal, taking the form of taxes, subsidies, material approvals, and eco-design standards [4,5]. Eco-design constitutes one of the components of circular economy implementation, and its role has been emphasized in the European Green Deal. The role of environmental performance measurement (as the main source of knowledge for eco-design) throughout the product life cycle is expected to increase in the coming years. Alongside the adoption of the EU’s circular economy targets, significant development of recycling [5] and waste recovery technologies is anticipated, with a focus on sorting materials that can be given a second life.

The construction sector is one of the fastest-growing industries in the economy. Tatara et al. [6] observe that an increasing importance is being attached to the use of innovative building materials (characterized by low heat transfer coefficients) and methods of reducing water and electricity consumption, thereby aiming to reduce the environmental pressure of this economic sector. The implementation of eco-solutions is particularly important due to the significant anthropogenic impact of construction on the environment [7,8], including the generation of increasing amounts of waste containing various types of stone aggregates and composite materials suitable for reuse [9,10,11]. According to Contreras-Llanes et al. [12] and Robayo-Salazar et al. [13], approx. one-third of all waste generated in the EU comes from construction and demolition waste (CDW).

CDW is defined as solid waste generated during construction, long-term operation (including renovations), and during the aging process of construction and finishing materials. It also results from demolition activities [7]. CDW primarily consists of mineral components such as crushed concrete, bricks, tiles, and asphalt, as well as plastics, wood, metals, glass, and cardboard [14].

The major problem in dealing with CDW is the optimal selection of methods for its management. Much of the CDW ends up directly in municipal waste landfills and illegal dumps [15]. Considering that construction materials are largely made from non-renewable raw materials, their reuse, e.g., as a substitute in construction-related production processes, is a necessary measure [16,17]. Plastics, ferrous and non-ferrous metals, wood not contaminated with hazardous substances, and, above all, stone aggregates of various grain sizes can be reused. Effective management of aggregates from CDW is a critical task due to the enormous economic significance of these materials [18]. The use of stone aggregates in construction and road building is constantly increasing; therefore, there is a continuous need to find natural stone aggregates—particularly waste materials—as substitutes obtained directly from the environment [19,20]. In addition to road construction, stone aggregates are also used in residential, commercial, industrial, and other infrastructure projects [21].

The need for higher efficiency of the waste recovery process is dictated by the European Parliament’s 2018 Waste Directive (Directive 2018/851). According to the Directive, the recovery rate of construction waste should have been 70% since 2020. Construction waste should be collected separately to increase the possibility of its reuse. Mixed construction waste generated in Europe can only be processed in specialized plants or using mobile equipment, such as crushers and sifters, directly at the place of waste generation (e.g., at a construction site). In this way, rubble, gypsum, and smaller stone aggregate fractions, including powder, are sorted from the waste and require further cleaning (e.g., sorting reinforcement from concrete structures). Large elements are separated manually, while small reinforcement components are separated using magnetic separators. Unfortunately, most functioning installations are limited to separating ferrous metals, fine fractions, and powders, with the remaining waste being deposited in landfills. Such installations are inadequate in the era of implementing the circular economy concept.

It is important to implement new, innovative technologies which allow for separating stone aggregates of different grain sizes and other materials that can be recycled (ferrous and non-ferrous metals) or recovered for energy (plastics, wood), and, above all, it is important to reduce landfilling of this type of waste. Unfortunately, separating 100% of recyclable materials from CDW is frequently unfeasible for technological or economic reasons [13].

In waste treatment processes (both municipal and industrial, including construction waste), increasing attention is being paid to environmental aspects and the implementation of sustainable production principles as well as the circular economy model. It is important in terms of non-financial reporting on sustainable development and resource conservation.

As indicated by Mesa et al. [22] and El-Shaboury et al. [23], the number of scientific studies analyzing the impact of waste recovery processes (including construction waste) is still insufficient to indicate the actual possibilities of using waste materials. The life cycle assessment (LCA) methodology is most often used to examine the environmental impact of various types of waste recovery processes [24,25]. The LCA methodology is also used to scale up developing technologies and to compare their potential with well-known technologies. This methodology enables the optimization of processes through ecodesign at an early stage of the decision-making process, which in the final effect can lead to an increase in the overall efficiency of the process and a reduction in costs.

In terms of CDW management, the LCA methodology has been applied to examine the efficiency of general CDW management strategies [26] and also to analyze the selected types of waste, e.g., photovoltaic panels [27], facade materials [28,29], windows [8], and many others. There are no studies covering the environmental impact of mixed CDW recovery facilities, particularly in terms of sorting recyclable materials. Conducting such an analysis using LCA offers a systematic comparison of alternative CDW recovery methods. The lack of such analyses limits the understanding of the relative environmental effects of different configurations of CDW recovery processes and underscores the novelty and importance of this study in filling this gap by providing reliable comparative data.

The research problem addressed in this study is to answer the question regarding the environmental impact of CDW treatment (taking into account factors such as electricity and fuel consumption, as well as the amount of waste generated and transferred to recycling, recovery, or disposal processes) and to identify those stages of the CDW recovery process that have the greatest positive or negative impact on the environment. The main objective of this study is to perform an environmental assessment of construction waste treatment (recovery) at three different facilities designed for this purpose.

## 2. Materials and Methods

LCA is a method for assessing the environmental aspects and potential environmental impact of products and processes. It was originally developed to assess the life cycle of a product, from natural resources exploitation, through processing, production, use, reuse, recycling, to final waste disposal, i.e., “from cradle to grave” or “from cradle to cradle” [25]. LCA is also used to assess the environmental aspects and impacts associated with various waste management scenarios. A systemic approach to life cycle assessment covers all areas of environmental impact related to waste management, including every waste processing procedure. Such an analysis is a tool allowing the assessment of different waste processing technologies characterized by varying electricity consumption patterns and production levels, as well as various material recovery rates.

The main goal of LCA is to provide a comprehensive insight into emissions to the environment and the use of natural resources caused by the waste management system or a waste management facility [30]. Conducting a life cycle assessment is a complex process requiring a large amount of highly precise data and methodologies for modeling environmental mechanisms and emission effects. Therefore, life cycle assessment is carried out using models specifically designed for this purpose [25]. LCA allows estimating the type and size of environmental threats generated by a product (its manufacturing, use, and waste management after exploitation) or a process during the successive stages, such as raw material acquisition, production, use, and waste management [31].

The LCA method was used in the presented analysis as its indirect goal most frequently targets the environmental impact assessment of a product or a process. LCA allows examining and comparing various scenarios, identifying problems and threats, and finally developing a model of planned improvements and their potential environmental impact [25]. Applying this method allows primarily for the identification of the process stages that exert the greatest environmental impact. LCA analysis comprises four main phases [32,33]: defining the purpose and scope of the analysis, conducting a life cycle inventory, assessing the life cycle impact, and interpreting the findings.

### 2.1. Goal and Scope, System Boundaries of Processes

The purpose of the conducted analyses was to identify the CDW processing technology (out of the three analyzed) that would achieve the highest environmental benefits while minimizing the negative environmental impact of the entire process. The scope of work included designing, assembling, and analyzing the three different technological systems for CDW recovery. The analysis of CDW recovery processes was performed for a functional unit consisting of 1 Mg of construction waste accepted for processing.

For each phase of the process, it was necessary to identify the resource flows entering and leaving the system. In this case, the primary resources used were electricity and diesel fuel for machines and transport, and the outputs consisted of generated wastes and the processes deployed for their final management. Capital goods and infrastructure were excluded, as recommended by ISO 14044 [34], since they do not affect the comparative results or ranking of the scenarios.

**Figure 1:**
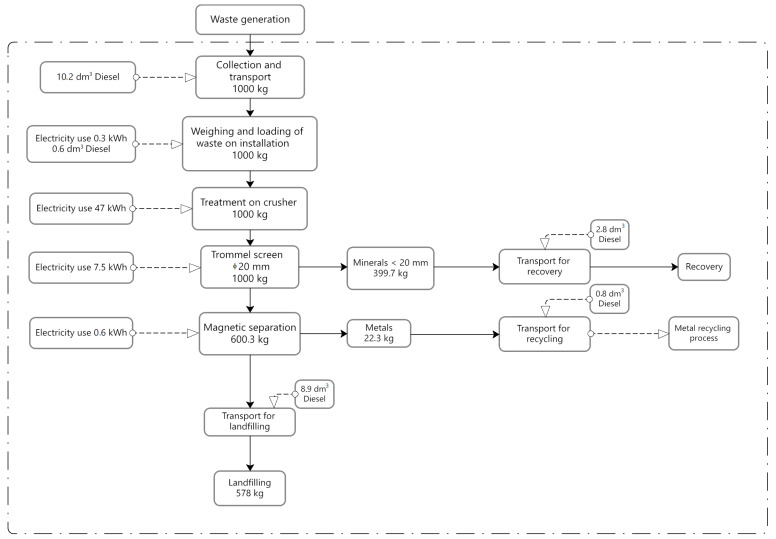
System boundaries for the process (installation) No. 1—basic. Source: Authors’ compilation.

**Figure 2:**
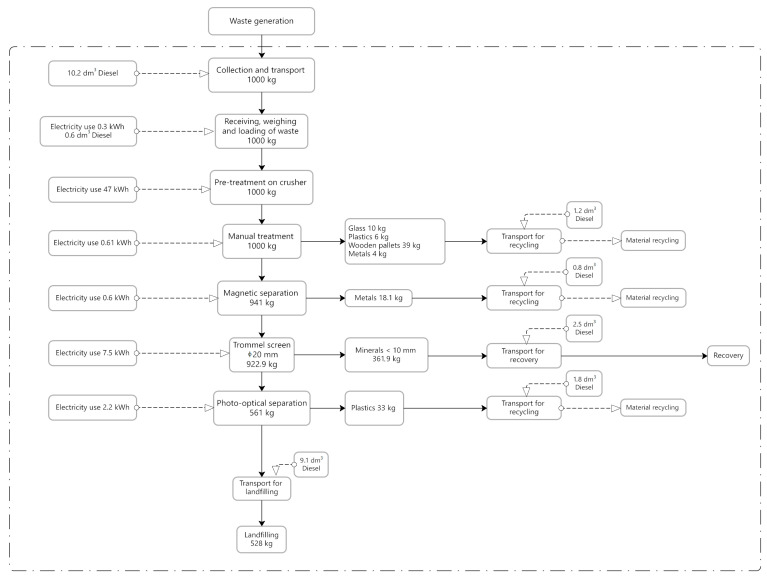
System boundaries for the process (installation) No. 2. Source: Authors’ compilation.

**Figure 3:**
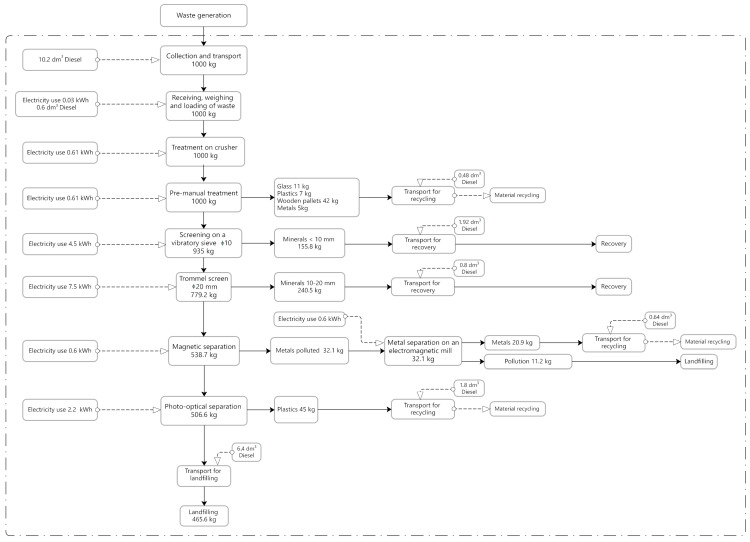
System boundaries for the process (installation) No. 3. Source: Authors’ compilation.

Individual process stages are presented in Figure 1, Figure 2 and Figure 3, in the form of block diagrams with marked system boundaries. These figures also present fuel and electricity consumption and the weight balance of processed waste. Data on material and energy consumption were obtained through direct measurements performed during the processes. Regarding energy, the electricity mix reflects the Polish market, as provided by official national sources and consistent with the Ecoinvent database.

Due to the purpose of this research, the system boundaries of the processes include only the recovery process that may take place at the waste management facility. This “gate-to-gate” approach is consistent for comparative studies with a focus on a specific stage of the life cycle. The system boundaries do not include the CDW generation process, but do include the end-of-life (EoL) of the product, as these phases differ depending on the processes used.

### 2.2. Description of Experiment

In order to achieve the objective of the study, three different technological systems were designed and constructed for CDW treatment, based on the equipment commonly used in construction waste management. Each installation, with an approximate capacity of 8 Mg/h, was subjected to a two-week analysis at a waste management company in Krakow (Poland). During the analyses, the electricity consumption of individual devices was recorded using the NP40 600MO network parameter analyzer produced by Lumel (Poland). Energy was modeled based on the Polish electricity mix (electricity, low-voltage (PL) market for the APOS System). It should be noted that Poland, where the analyses were conducted, still relies heavily on hard coal and lignite, which significantly increases the carbon footprint of electricity compared with countries dominated by renewable energy sources or nuclear power.

Fuel consumption (actual diesel consumption for individual machines) was also monitored using on-site refueling records. Additionally, the amount of waste accepted for processing and the amount of waste generated, broken down by individual fractions, were measured using a 40 Mg certified weighbridge with a measurement accuracy of ±10 kg. The study also considered the average transportation distances for waste to the processing facilities and from these facilities to subsequent management sites (recycling, recovery, or disposal-landfilling). In the analysis, it was assumed that recovered materials are reallocated: recycled steel substitutes primary steel, recycled plastic substitutes polyethylene and polypropylene, recovered wood substitutes pellet or sawn timber, and recycled aggregates substitute gravel or natural stone.

During the period under study, an average of 1250 Mg of CDW was processed at each of the three facilities as part of the conducted analyses. To ensure comparability of the input streams among the analyzed installations, the CDW samples had similar material composition, originated from the summer period (July/August), from the same region, and exhibited similar moisture content (16.1 ± 1.7%), as well as comparable levels of organic and hazardous contaminants.

Mixed construction and demolition waste was collected for analysis from households and construction companies operating in Krakow (Poland) and the surrounding municipalities. The composition of waste accepted for processing was determined based on 24 unit samples, of 2 m^3^ volume each. For each of the installations under study, 8 samples were collected. The unit sample was prepared using the quartering method presented by Famielec et al. [2]. The composition of the material was obtained by dividing this waste into 13 different fractions. The separation was carried out using a multi-level 10 and 20 mm mesh sieve and manual segregation. The weight was measured on a certified platform scale with a maximum capacity of 300 kg and a measurement accuracy of ±0.1 kg. Performing these analyses was necessary to determine the percentage of CDW suitable for recycling and recovery.

The first installation analyzed was the most common CDW sorting machine system in Europe and consisted of an impact crusher (Komatsu, Tokyo, Japan), a mobile trommel screen with a mesh diameter of Ø 20 mm (Doppstadt SM620, Calbe, Germany), and a magnetic separator (Magnetix STM, Toruń, Poland). The installation elements were connected by belt conveyors with a power of 11 kW and a width of 1.1 m. Waste was loaded into the installation using a backhoe loader (JCB 457 WLS, Staffordshire, UK). The recovery process separated minerals, i.e., stone aggregates of up to 20 mm grain size, including powder. In addition, ferrous metals were separated on a magnetic separator. The waste accepted for processing was transported from an average distance of 28 km. Transportation was carried out using a heavy commercial vehicle (Transport, Freight, lorry > 32 metric ton, EURO5, APOS, System). The separated minerals were transported approx. 10 km for reuse, while the metals were sent for recycling. The remaining waste, i.e., plastics, paper, and glass, was transferred as mixed waste to a landfill.

The second installation consisted of the same impact crusher, a sorting cabin (EDGE, Poznań, Poland) for manual sorting on belt conveyors, a magnetic separator, a mobile trommel screen with a mesh size of Ø 20 mm, and one photo-optical separator (Tomra, Warszawa, Poland) for separating plastic (white and colored film). During waste processing, the following materials were separated manually: glass, plastics, wooden pallets, metals, which were sent for recycling, and minerals (fine stone aggregates and powder), which, as in the case of the first installation, were sent for reuse. The waste was transported from an average distance of 28 km.

The third installation consisted of the following components: the same impact crusher, a sorting cabin with belt conveyors, a vibrating screen (Luxor, Lublin, Poland) with a maximum mesh size of Ø 10 mm, a mobile trommel screen with a mesh size of Ø 20 mm, an electromagnetic mill (Eltraf, Lubliniec, Poland), and a magnetic and photo-optical separator (previously used at installation No. 2). The installation separated glass, plastics, wooden pallets, and metals transported to recycling plants, as well as minerals sent for reuse.

Detailed technological diagrams, including fuel and electricity consumption and also the weight balance of processed waste for each of the above-described CDW recovery process variants, are presented in Figure 1, Figure 2 and Figure 3. None of the analyzed installations extracted hazardous waste (such as paint and varnish containers) or organic waste. Many contaminants, such as construction polystyrene, tires, and organic waste (grass), were left in the waste stream. Separating them would require increasing the number of employees at the installation and a higher share of human labor.

During processing, at each installation, there were some losses in the waste mass, mainly due to moisture losses (evaporation of water from the waste) and dust emissions, which were captured by bag filters. The losses were less than 0.1% of the processed waste.

### 2.3. Life Cycle Impact Assessment and Interpretation

In this study, SimaPro 8.1 software (PRé Sustainability BV, Amersfoort, The Netherlands) and the Ecoinvent v3.8 database were used to analyze the environmental impact of the process. The Ecoinvent database currently provides well-documented data processing for thousands of products [35]. It presents the defined impacts of various substances and processes on soil, water, and air pollution within the defined limits. In the LCA study, an attributional allocation approach (APOS) was applied. This approach distributes environmental burdens between co-products based on their physical relationships and recycled content, and is widely used in comparative LCA studies. The ReCiPe 2016 v.1.1 method was used, which allows for the environmental impact assessment at two levels: Midpoint (Hierarchist) and Endpoint. The midpoint visualization provides greater scientific robustness and lower uncertainty. The hierarchical approach includes effects in the mid-term time horizon.

ReCiPe was developed in 2008 in collaboration between RIVM, Radboud University Nijmegen, Leiden University, and PRé Sustainability. This method is one of the most widely applied, according to the literature [2,7,8]. The main goal of the ReCiPe model is to convert a long list of life cycle inventory results into a limited number of indicators. The ReCiPe defines indicators at two levels: 18 midpoint indicators and three endpoint indicators. The ReCiPe Midpoint (H) method is used to assess environmental impacts. The Midpoint method analyzes the impact of previous cause-and-effect chains before reaching the endpoint. The endpoint approach is focused on analyzing environmental impacts from the last cause-and-effect chain. Midpoint indicators represent indirect measures of environmental impact that reflect changes in the natural environment caused by emissions or resource consumption to show where each impact category is characterized. The ReCiPe includes three endpoints (human health, ecosystem, and resources) [36].

At the endpoint level, most of these middle impact categories are multiplied by harm factors and aggregated into three endpoint categories: human health, ecosystems, and resources. The endpoint characteristics used in ReCiPe can be described as follows [37]:

1. Human health is expressed as the number of years of life lost and the number of years lived with disability. They are combined as disability-adjusted life years (DALY), an indicator also used by the World Bank and the WHO.
2. Ecosystems are expressed as the loss of species in a given area over a given time.
3. Resource scarcity is expressed as the excess cost of future resource production over an infinite timeframe (assuming constant annual production), taking into account a 3% discount rate. It should be remembered that fossil resource scarcity does not have a constant mid-to-endpoint ratio, but rather individual ratios for each substance.

In the ReCiPe model, the output unit is the total environmental load point, the so-called eco-point (Pt), which corresponds to a unit known as personal equivalent (PE). A unit of 1000 PE represents the annual environmental impact caused by all the activities of an average European [37].

Additionally, a sensitivity analysis was carried out, which is particularly relevant before up-scaling. It is used to evaluate how impacts change if a variable is changed. The choice to modify the energy source is justified because this is the component that can most influence the results. It was assumed that solar energy could be used to operate all the equipment through the use of photovoltaic (PV) panels. Consequently, the electricity from the Polish energy mix is replaced in 20% with renewable energy from PV panels (Electricity, low voltage, electricity production, photovoltaic, 3 kWp, slanted-roof installation, multi-Si panels, mounted, APOS, System).

Moreover, the results present the outcomes of hypothetical calculations concerning the environmental impact of the analyzed installations, excluding the most negatively affecting stages. The environmental impact of a zero-scenario was also examined, i.e., a scenario in which construction and demolition waste would not be processed and would be disposed of directly (without treatment) in a municipal landfill.

## 3. Results

### 3.1. Material Composition of Waste

The problem of mixed CDW treatment results primarily from its highly diverse material composition (Table 1). Concrete rubble dominates this waste stream, while, in terms of volume, materials such as wood, mineral wool, plastic film, other plastics (e.g., polystyrene), and paper packaging predominate. However, these materials (polystyrene, wool, plastic film) are much lighter than rubble. Plastic film (often of large surface), polystyrene, metals (metal rods and fittings), as well as wood (pallets, boards, panels, chipboard, etc.) are particularly noticeable in the morphology of this waste, and they pose the greatest challenge in terms of construction waste recovery. The analyzed waste also contained food residues, as well as soil and reinforced concrete slabs, which are characteristic of road construction debris. Due to the presence of slabs, a crusher was an indispensable element of each of the designed installations. The recycling of construction and demolition waste should be focused on extracting (separating) the components to be used as raw materials in other technological processes and on separating stone aggregates of different grain sizes. The material analysis (Table 1) shows that over 16% is suitable for recycling; however, sorting such a large amount of material fractions turns out to be impossible due to their high degree of contamination. Approx. 60% of the waste (mainly concrete, brick, and other debris) is suitable for the production of stone aggregates, but, as in the case of secondary raw materials, it is also impossible to sort such a mass of waste due to its exposure to contamination.

### 3.2. Material Recovery Results

The analyzed processes differed significantly in terms of electricity and fuel consumption (Figure 1, Figure 2 and Figure 3). Each subsequent technological process involved the addition of further machines and equipment, which mainly resulted in increased electricity consumption. Type 1 installation presented the lowest electricity and fuel consumption, whereas Type 3 installation (process) had the highest, with the impact crusher being responsible for the highest electricity consumption in each case. All of the analyzed facilities were characterized by different results in terms of the sorted waste weight and subsequently transferred for recycling (materials), recovery (stone aggregates), and landfill, respectively. Table 2 presents the results related to CDW recovery at each of the analyzed facilities. These are empirical results obtained under Polish conditions for a specific stream of waste originating from the Kraków region, which has its own local CDW morphology. The yields may differ in other countries due to differences in the structure of construction waste, the technologies available, and the culture of waste sorting.

According to the assumptions, the machines and equipment used in Type 3 installation enabled the extraction of over 13% of the available material and raw material fractions. In addition, approx. 47% of the received waste was transferred to the landfill as part of this process. In contrast, the Type 1 process (basic version) involved only metals being sent for recycling, with approximately 58% of the entire waste stream subject to processing being disposed of in the landfill.

### 3.3. Environmental Assessment

**Figure 4:**
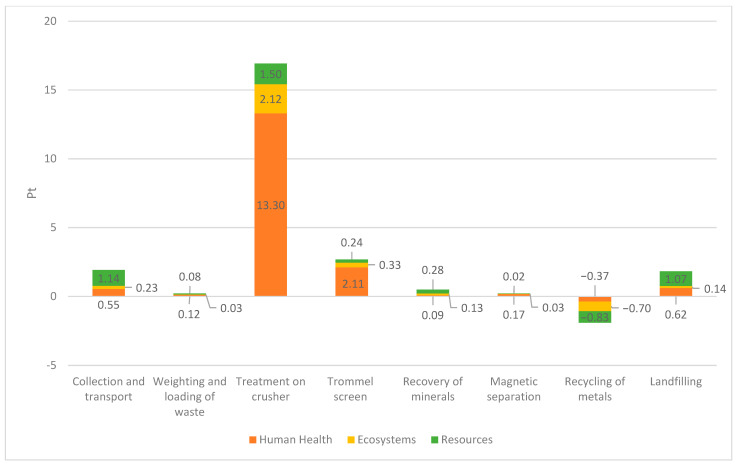
Comparison of the environmental impact of individual stages of the construction and demolition waste recovery process at installation No. 1. Source: Authors’ compilation.

Figure 4 illustrates the environmental impact of individual stages for Type 1 CDW recovery installation expressed in eco-points (Pt). The environmental analysis of the first analyzed installation showed that the treatment process involving a crusher (16.92 Pt) has the most negative impact on the environment. Other elements of the system, such as collection and transport (1.92 Pt), trommel screen (2.68 Pt), and landfilling (1.83 Pt), also influence the environment negatively, but to a much lesser extent than crushing. Only the metal recycling stage (−1.90 Pt) has a positive impact on the environment in the analyzed process.

**Figure 5:**
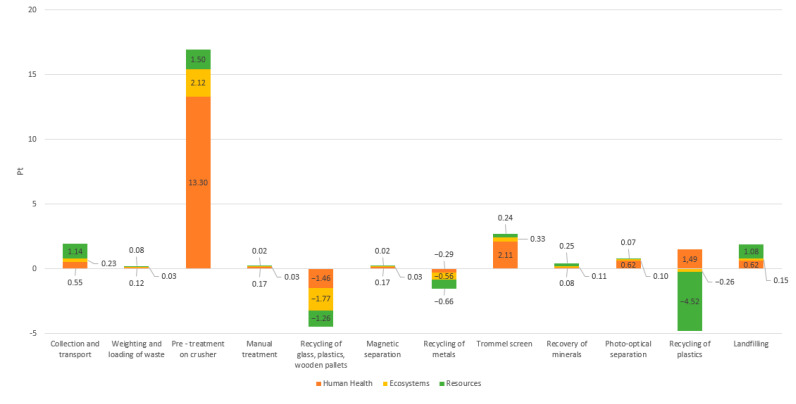
Comparison of the environmental impact of individual stages of the construction and demolition waste recovery process at installation No. 2. Source: Authors’ compilation.

Figure 5 presents the results of the environmental analysis for the second installation, for which the crusher stage treatment has the most negative impact on the environment, similarly to the results for Type 1 installation. The stages which also exert a negative impact on the environment in the discussed process are as follows: collection and transport (1.92 Pt), trommel screen (2.68 Pt), and landfilling (1.85 Pt). Installation No. 2 performs significantly more process stages than installation No. 1. Manual treatment (0.22 Pt), photo-optical separation (0.79 Pt), and magnetic separation (0.22 Pt) involve electricity and fuel consumption, but ultimately, the transfer of sorted materials for recycling results in positive environmental changes, mainly related to resource conservation (recycling of materials sorted in the sorting cabin: −4.49 Pt; recycling of metals sorted on the magnetic separator: −1.51 Pt; recycling of materials sorted on the photo-optical separator: −3.28 Pt).

**Figure 6:**
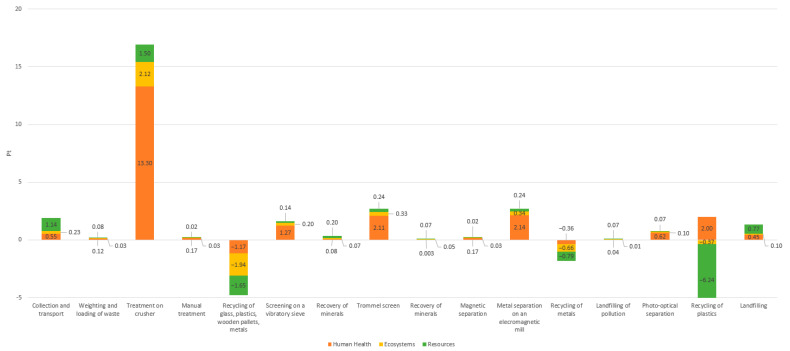
Comparison of the environmental impact of individual stages of the construction and demolition waste recovery process at installation No. 3. Source: Authors’ compilation.

The most complex technological process among the analyzed ones was carried out at installation No. 3 and consisted of a total of nine processing stages. A comparison of the environmental impact of each individual stage is presented in Figure 6. In this case, the crushing is also the stage exerting the most negative impact on the environment. Other processes that have a negative impact on the environment include: metal separation on an electromagnetic mill (2.72 Pt), collection and transport (1.92 Pt), trommel screen (2.68 Pt), landfilling of waste sorting residues (1.32 Pt), and screening using a vibratory sieve (1.61 Pt). A positive impact on the environment is associated with the recycling of materials that have been separated during manual treatment (−4.76 Pt), magnetic separation (−1.80 Pt), and photo-optical separation (−4.61 Pt).

**Figure 7:**
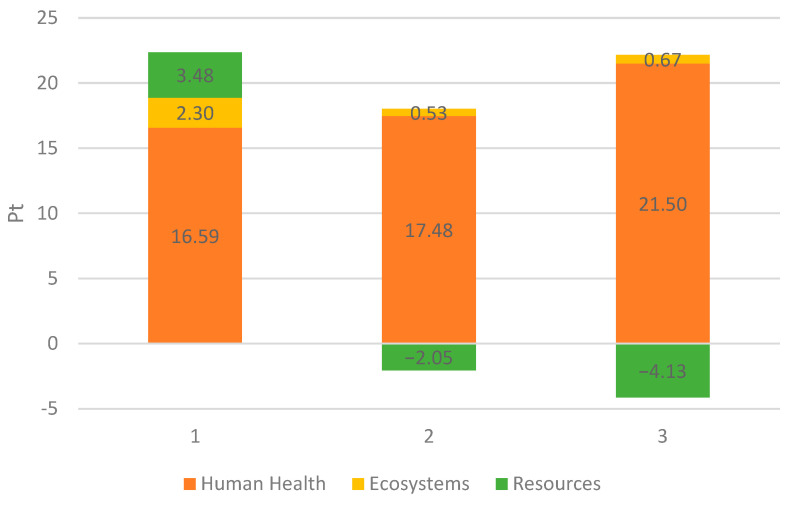
Comparison of the environmental impact of the analyzed waste recovery processes. Source: Authors’ compilation.

Figure 7 provides a graphical summary of the environmental impact of the three analyzed CDW recovery processes. The total negative environmental impact for installation No. 1 is 22.37 Pt, for process No. 2—15.96 Pt, and for installation No. 3—18.04 Pt. If only electricity and fuel consumption were taken into account, the installation No. 3 would be responsible for the greatest damage to the environment (as evidenced, i.e., by the high damage calculated in the Human Health category). Sorting waste, which was then sent for recycling, reduced the environmental impact in terms of ecosystems and resources, and this ultimately resulted in installation No. 2 offering the lowest total impact on the environment, even though the largest amount of recyclable materials was sorted at installation No. 3.

Table 3 presents a summary of emissions to the environment for the three analyzed processes, developed based on the ReCiPe Midpoint (H) methodology. Process No. 1, despite having the lowest impact in 8 of the 18 analyzed categories, including ozone depletion, freshwater eutrophication, and photochemical oxidant formation, is also characterized by extremely high values in key areas such as climate change (71.53 kg CO_2_ eq) and fossil resource depletion (40.26 kg oil eq), which significantly lowers its overall environmental rating and disqualifies it as a sustainable option. Process No. 1 scored favorably in some impact categories due to its relatively low energy demand. However, its overall performance was the worst among the three facilities. This apparent paradox is explained by two factors: first, the low recovery rates translate into limited recycling credits as only small amounts of aggregates and metals are substituted; second, the categories of climate change and fossil resource depletion are dominant in the total score, where process No. 1 performs poorly because the avoided burdens are negligible while diesel use and dependence on the Polish electricity mix remain significant. Despite its efficiency in terms of direct electricity consumption, installation No. 1 offers limited environmental benefits from a system perspective.

Process No. 3 performs well in selected categories, achieving the lowest values in, i.e., climate change (17.64 kg CO_2_ eq), and also showing negative values in 4 out of 18 categories, such as metal depletion (−22.99 kg Fe eq) and fossil resource depletion (−28.61 kg oil eq), which is related to the recycling of materials recovered from the waste stream (mainly metals, wood, and plastics). Nevertheless, the clearly negative impact in areas such as human toxicity, ionizing radiation, and water depletion limits its overall positive assessment as an environmentally beneficial solution.

Against this background, process No. 2 proves to be the most sustainable and environmentally optimal solution. Compared to process No. 1, it reduces climate change by as much as 73.8% and fossil resource depletion by over 130%. On the other hand, compared to process No. 3, it generates a 20% lower impact on human toxicity, 26% less water depletion, and over 21% lower ionizing radiation. Importantly, process No. 2 shows negative values in as many as 5 out of 18 categories, including agricultural land occupation (−45.75 m^2^a), urban land occupation (−0.04 m^2^a), metal depletion (−16.42 kg fe eq), and fossil resource depletion (−12.02 kg oil eq). The process carried out at installation Type 2 achieved the best overall environmental balance. Although it does not dominate in the number of the best unit indicators, it consistently maintains low and stable values in all areas and demonstrates potential for a positive impact on the environment.

Negative results and very low values in Table 3 arise from material recovery and the associated allocation: for example, recycled steel replacing primary steel (BOF route), recycled plastic replacing virgin polyethylene/polypropylene, recovered wood replacing pellet or sawn timber, and recovered aggregates replacing natural gravel/stone. In this way, recovery offsets part of the impacts, subtracting them from the balance, which yields negative or very low values. The same phenomenon was shown in studies by Koinig et al. [38], Pešta et al. [39], and Mazzi et al. [40].

### 3.4. Sensitivity Analysis

In each of the analyzed installations, the greatest environmental impact was represented by the crusher. If the use of the crusher were to be abandoned (which is not currently feasible in the CDW recovery process), the environmental impact of installations No. 1, No. 2, and No. 3 would be 5.45 Pt, −0.95 Pt, and 1.12 Pt, respectively. However, such solutions should be treated as hypothetical. Importantly, not processing CDW and directing it directly to a landfill results in a negative impact of 3.1 Pt, which is higher than for installations No. 2 and No. 3.

Table 4 shows the comparison between the environmental performances of the three processes, using the 20% needed energy from renewable energy sources (photovoltaic panels (PV)). The use of energy from photovoltaics is preferable for most impact categories.

## 4. Discussion

Research conducted in many countries around the world indicates that construction waste is one of the most commonly discarded material fractions in illegal landfills [15,41]. Such practices result not only in the loss of material resources but, above all, in a number of undesirable effects on the environment [41]. Laws, directives, and guidelines on waste management require selective collection and recovery, yet much of this waste ends up in landfills. In addition, numerous barriers, such as a lack of public awareness, insufficient enforcement of regional and national policies, and the absence of economic incentives, hinder the development of infrastructure for recycling CDW in many developing countries [15,41]. Rosado et al. [42] emphasize that only the treatment of CDW in facilities adapted for this purpose allows environmental benefits to be achieved. These benefits mainly come from sorting metals, wood, and plastics for recycling. Similarly, Chen et al. [43], who conducted a systematic review of 46 studies on CDW upcycling, emphasize the dominant role of physical processing technologies and their impact on air pollution emissions and climate change.

The conducted analyses have shown that, under Polish conditions, it is possible to recover approx. 13% of materials (for recycling) and approx. 40% of waste for reuse in the construction process from CDW as a result of their treatment at dedicated installations. As a result, these materials will not end up in illegal landfills and will avoid storage, thus generating a positive impact on the environment. In the countries prone to earthquakes (e.g., Turkey), the topic of CDW recovery is also widely discussed. Bilgili and Çetinkaya [44] assessed various recovery scenarios in the context of post-earthquake waste management, indicating that scenarios with maximum recovery of construction materials (e.g., concrete) are significantly more environmentally beneficial than the mixed or landfill-dominated scenarios.

Despite legislative changes in Poland, a large amount of CDW still ends up in landfills, despite the obligation to recover it, in the context of implementing a circular economy. The comparative analysis indicates that the environmental performance of the local CDW processing system aligns with international experience, where high recovery rates, mechanized sorting, and optimized recycling are essential to minimize environmental impacts. Our results are consistent with observations from countries such as Germany, Austria, and the Netherlands, where advanced sorting technologies and targeted recycling of construction materials significantly reduce resource depletion and impacts on human health.

A significant limitation of the conducted analyses was the use of emission factors from fuel combustion and electricity generation under Polish conditions. The authors are aware that applying indicators provided by the Ecoinvent database for other countries characterized, e.g., by a different energy mix, may yield different results.

Banias et al. [45] conducted the analysis covering three CDW management strategies for Greece, i.e., recycling, recovery, and landfilling, indicating that hybrid systems (combining recovery and recycling) provide the best environmental balance. In addition, it was found that the entire CDW management process can be further improved through applying building information modeling (BIM). Iodice et al. [46], in a study addressing the Italian CDW management system, showed that selective dismantling increases the recovery rate up to 90% along with reducing the impact on human health by 27%, as compared to the standard scenario.

Our study confirmed that fuel combustion and electricity consumption have the greatest negative impact on the environment, which translates into damage in the human health category. Gálvez-Martos et al. [47] showed that using mobile CDW crushing units, although more flexible in terms of operation, leads to higher PM10 emissions and fuel consumption compared to stationary units. Dierks et al. [48], studying the environmental impact of low- and high-quality concrete waste recycling in Germany, found that greenhouse gas (GHG) emissions are mainly associated with stationary concrete waste treatment, including crushing and sorting. It was observed that the demand for electricity in these processes is the main factor resulting in GHG emissions. On-site transport using excavators contributes to a much lesser extent to GHG emissions, highlighting the negative impact of the crushing stage on the environment. It is important to note that, based on the conducted analysis, the crushing process also played a dominant role in the environmental impact. In the studies carried out by Bilgili and Çetinkaya [44], it was found that the use of heavy combustion equipment causes up to four times higher emissions into the environment.

When reviewing the results of various studies, one may come across findings indicating that CDW processing is not always the optimal solution from a life cycle assessment perspective. Atta and Bakhoum [49] demonstrated that CDW recycling processes generate higher PM_2.5_ emissions compared to natural stone aggregate extraction, mainly due to emissions from diesel engines during demolition and transport. Although PM_10_ emissions were lower in the case of recycling, the overall impact on human health was greater in the recycling scenario. The authors of this study indicate that the distance of 70 km over which waste is transported determines its cost-effectiveness from a life cycle perspective [49]. Similarly, Gálvez-Martos et al. [47] demonstrated that optimizing CDW transport logistics can reduce greenhouse gas emissions by up to one-third.

Zhao et al. [50], studying the environmental benefits of using stabilized recycled CDW aggregates in the context of life cycle costs, observed a significant reduction in SO_2_ and CO_2_ emissions. Mesa et al. [22] also point to a reduction in SO_2_ emissions of up to 70% in CDW recycling processes as compared to conventional materials. The benefits of using recycled materials from CDW are also highlighted by researchers involved in recycling these materials. The processing of steel and aluminum from demolition leads to significant reductions in CO_2_ and SOₓ emissions compared to primary production. For aluminum, this reduction can be as high as 95.8% [51].

## 5. Conclusions

The environmental LCA assessment conducted for the construction waste recovery processes resulted in the following conclusions:

- The CDW recovery process has a negative impact on the environment, mainly due to fuel and electricity consumption, which is consistent with the LCA results, particularly in terms of climate change, human health, and fossil resource depletion.
- The most burdensome stage is waste crushing (treatment involving a crusher), which is consistent with the results for all three analyzed installations. This stage is a critical link in terms of the environment. In subsequent analyses, the possibility of discontinuing this device usage within certain limits should be considered.
- Materials sorted during manual, photo-optical, and magnetic sorting have an overall positive impact on the environment, mainly due to resource savings.

## Figures and Tables

**Table 1:** Material composition of mixed construction and demolition waste (mean ± SD; n = 24).

No.	Type of Waste	Mass Fraction of Contaminants [%]
1	Brick rubble	11.4 ± 6.8
2	Concrete rubble	30.8 ± 6.3
3	Other rubble waste (hollow bricks, crushed stone, ceramics, etc.)	18.7 ± 5.9
4	Gypsum plasterboards	8.1 ± 4.8
6	Wood (construction and packaging)	4.9 ± 4.0
7	Metals	3.2 ± 2.2
8	Plastics—mainly plastic film, construction polystyrene, and mineral wool	5.9 ± 3.1
9	Paper and cardboard packaging (including bags for adhesives, cement, and mortar)	2.6 ± 1.1
10	Construction and packaging glass	5.2 ± 2.1
11	Soil	1.6 ± 0.8
12	Hazardous waste (packaging from paints and varnishes)	0.1 ± 0.1
13	Other (including textiles, organic materials, electronics, roofing felt, etc.)	7.5 ± 2.4

Source: Authors’ compilation based on own research.

**Table 2:** Utilization of sorted materials (mean ± SD).

No.	Process	Unit	Share of Materials Sent for Recycling	Share of Materials Sent for Further Recovery	Share of Waste Sent to Landfill
1	Installation 1 (n = 10)	%	2.2 ± 0.9	39.9 ± 3.1	57.9 ± 3.9
2	Installation 2 (n = 10)	%	11.0 ± 1.3	36.2 ± 4.4	52.8 ± 4.6
3	Installation 3 (n = 10)	%	13.1 ± 1.4	39.6 ± 4.2	47.3 ± 4.8

Source: Authors’ compilation based on own research.

**Table 3:** Summary of emissions to the environment from individual processes.

No.	Impact Category	Unit *	Process 1	Process 2	Process 3
1	Climate change	kg CO_2_ eq	71.52966	18.7309	17.6384
2	Ozone depletion	kg CFC-11 eq	1.36 × 10^−5^	1.56 × 10^−5^	1.49 × 10^−5^
3	Terrestrial acidification	kg SO_2_ eq	0.402112	0.259968	0.29452
4	Freshwater eutrophication	kg P eq	0.066238	0.078773	0.096067
5	Marine eutrophication	kg N eq	0.019783	0.032948	2.80 × 10^−5^
6	Human toxicity	kg 1,4-DB eq	44.18279	51.55441	64.80092
7	Photochemical oxidant formation	kg NMVOC	0.195164	−0.12266	−0.1898
8	Particulate matter formation	kg PM10 eq	0.060619	0.028752	0.019776
9	Terrestrial ecotoxicity	kg 1,4-DB eq	0.002491	0.000443	0.002971
10	Freshwater ecotoxicity	kg 1,4-DB eq	1.133928	1.378836	1.766191
11	Marine ecotoxicity	kg 1,4-DB eq	1.080263	1.298984	1.663928
12	Ionizing radiation	kBq U235 eq	6.684065	13.53276	17.19614
13	Agricultural land occupation	m^2^a	2.320457	−45.7499	−24.8917
14	Urban land occupation	m^2^a	0.509066	−0.03988	0.457044
15	Natural land transformation	m^2^	0.017242	0.020657	0.023887
16	Water depletion	m^3^	0.210985	1.416365	1.902959
17	Metal depletion	kg Fe eq	−17.9626	−16.4212	−22.9917
18	Fossil resource depletion	kg oil eq	40.26342	−12.0228	−28.6057

* CFC—the characterization factor for ozone layer depletion accounts for the destruction of the stratospheric ozone layer by anthropogenic emissions of ozone depleting substances, 1,4-DB—the characterization factor of human toxicity and ecotoxicity accounts for the environmental persistence (fate) and accumulation in the human food chain (exposure) and toxicity (effect) of a chemical, NMVOC—the unit of human health ozone formation potential; the characterization factor is determined from the change in intake rate of ozone due to change in emission of precursors, and kBq U235—the characterization factor of ionizing radiation accounts for the level of exposure for the global population [36]. Source: Based on own research.

**Table 4:** Results of the sensitivity analysis of the processes.

No.	Impact Category	Unit	Process 1	Process 2	Process 3
1	Climate change	kg CO_2_ eq	68.00209	14.02327	9.910011
2	Ozone depletion	kg CFC-11 eq	1.36 × 10^−5^	1.56 × 10^−5^	1.49 × 10^−5^
3	Terrestrial acidification	kg SO_2_ eq	0.384152	0.236	0.255511
4	Freshwater eutrophication	kg P eq	0.062421	0.073678	0.088407
5	Marine eutrophication	kg N eq	0.018783	0.031615	−0.00202
6	Human toxicity	kg 1,4-DB eq	41.87031	48.46835	60.25352
7	Photochemical oxidant formation	kg NMVOC	0.187939	−0.1323	−0.20584
8	Particulate matter formation	kg PM10 eq	0.055455	0.02186	0.007485
9	Terrestrial ecotoxicity	kg 1,4-DB eq	0.002748	0.000786	0.003683
10	Freshwater ecotoxicity	kg 1,4-DB eq	1.108084	1.344347	1.739121
11	Marine ecotoxicity	kg 1,4-DB eq	1.053006	1.262609	1.632064
12	Ionizing radiation	kBq U235 eq	6.609716	13.43354	17.04824
13	Agricultural land occupation	m^2^a	2.201047	−45.9092	−25.1855
14	Urban land occupation	m^2^a	0.494737	−0.059	0.4279
15	Natural land transformation	m^2^	0.017145	0.020527	0.023563
16	Water depletion	m^3^	0.210539	1.41577	1.861837
17	Metal depletion	kg Fe eq	−17.8968	−16.3335	−22.8058
18	Fossil resource depletion	kg oil eq	39.32948	−13.2692	−30.7083

Source: Based on own research.

## Data Availability

The original contributions presented in this study are included in the article. Further inquiries can be directed to the corresponding author.

## Abbreviations

The following abbreviations are used in this manuscript:

CDW	Construction and demolition waste
LCA	Life cycle assessment
WHO	World Health Organization
PE	Personal equivalent
